# Difference of ecological half-life and transfer coefficient in aquatic invertebrates between high and low radiocesium contaminated streams

**DOI:** 10.1038/s41598-020-78844-8

**Published:** 2020-12-11

**Authors:** Mayumi Yoshimura, Akio Akama

**Affiliations:** 1 Forestry and Forest Products Research Institute, Kansai Research Center, Nagaikyutaro 68, Momoyama, Fushimi, Kyoto 6120855 Japan; 2 Forestry and Forest Products Research Institute, Matsunosato 1, Tsukuba, Ibaraki 305-8687 Japan

**Keywords:** Ecology, Ecology, Environmental sciences, Limnology, Natural hazards

## Abstract

The Fukushima accident emitted radioactive substances into the environment, contaminating litter, algae, sand substrate, aquatic invertebrates, and fish in freshwater streams. Because these substances have substantial effects on stream ecology over many years, it is necessary to clarify the diffusion and decay mechanisms of radiocesium. The transfer coefficient differed among aquatic invertebrate groups, likely due to the differences in habitat. The ecological half-life of cesium was longer where the air dose rate was lower. The transfer coefficient was also higher in areas with lower air dose rate. The radiocesium concentration in algae was inversely related to stream current velocity in the radiocesium-contaminated area. However, this relationship was not observed in the lower air dose rate area: the radiocesium concentration in algae in the rapid-velocity areas tended to be higher than that in the slow-velocity areas. This reverse trend would lead to a longer period of freshwater contamination. The radiocesium concentration would continue to decrease in highly contaminated areas, but it would be difficult to reduce the radiocesium concentration in less-contaminated areas because different contamination mechanisms are at work. Controlling the water flow is key to regulating radiocesium concentration in freshwater ecosystems.

## Introduction

The earthquake on March 11, 2011 in eastern Japan, triggered a tsunami that washed over the Fukushima Daiichi nuclear power plant (FDNPP) on the eastern coast of Japan. Damage to the cooling system of the FDNPP resulted in several explosions, which caused leakage of the radioactive substances. This accident released 1.6 × 10^17^ Bq of iodine-131, 1.8 × 10^16^ Bq of cesium-134 and 1.5 × 10^16^ Bq of cesium-137 into the surrounding environment^1^. Most of these radionuclides, including iodine-131, cesium-134 and cesium-137, spread over large areas of land in eastern Japan, and reached sites located hundreds of kilometres from the FDNPP. Atmospheric dose rates at 0.5 m above the ground exceeded 20 μSv/h at some hot spots > 160 km from the FDNPP in May 2011^2^.

Early investigations revealed that a large amount of the cesium-134 and cesium-137 released from the FDNPP was trapped in the forest canopy and litter layer on the forest floor^3^. Cesium-137 is readily adsorbed by clay minerals and organic matter in soil^4^ and can be transported by eroding soils and as dissolved organic matter via hydrological channels to streams^5–7^. Cesium-137 adsorbed to particles would then be trapped in the microbial and algal mats^8^. Dissolved cesium-134 and cesium-137 in running water that is not adsorbed into the soil and particles is readily taken up by microbes, algae, plankton and plants. These transport pathways of cesium-134 and cesium-137 will eventually lead to uptake by aquatic invertebrates and freshwater fishes at higher trophic levels in the food web^5,9–11^.

The Chernobyl accident released more than 5 × 10^18^ Bq of radionuclides. Snowmelt water deposits cesium-134 and cesium-137 onto the dry riverbeds of streams, leading to the contamination of freshwater ecosystems. In the Dnieper River floodplain ecosystem, 85–97% of cesium-137 was accumulated in aquatic plants, 1–8% in zoobenthos, 1–8% in fish and 1% in gastropod molluscs^12^. Five to nine years after the accident, cesium-134 and cesium-137 levels had fallen in the lake water, but radioactivity levels increased in the sediments^12^. The broad effects of the Chernobyl accident were examined and management measures were subsequently designed for large geographical areas^12,13^. However, there are few studies focusing on radioactive contamination in freshwater biota such as algae and aquatic invertebrates.

Differences in the stream substrate structure play a large role in the degree of habitat contamination. Aquatic insects in stagnant pools generally exhibit higher radiocesium levels than those in flowing water, although the extent of cesium contamination in sand substrates do not significantly differ between pool and riffle sites^10^. Most of the radiocesium in ponds is in the sediment, and aquatic insects in the pond acquire radionuclides from the sediment through ingesting biotic components^11^. Aquatic insects inhabiting pools thus ingest high levels of radiocesium from the material settling at the bottom. Therefore, aquatic insects that can swim quickly and move between riffles and pools frequently, such as members of Ameletidae^12^, exhibit no differences in their radiocesium contents whether they are found in riffles or pools^10^. Considering the higher levels of contamination in aquatic insects in pools, the radiocesium concentration in algae attached to stones in the pool, which constitutes the diet of some aquatic insects, should therefore also be higher than that of algae in the riffles.

Chernobyl accident spread many radionuclides over a 2000 km from the pollution source, such as Finland, Sweden and Norway. Extensive cesium contamination in pools due to the Chernobyl fallout has also been detected in Norway^16^. Fish have been monitored for radioactivity in Øvre Heimdalsvatn, a Norwegian subalpine lake over the course of two decades^17,18^. Concentrations of cesium-137 in brown trout in the lake reached 8400 Bq/kg in 1987 and declined to 200–300 Bq/kg in 2008. Recently, the contamination level has remained low and is declining in an asymptotic manner^18^. However, the ecological consequences of this type of radioactive contamination in stream ecology are poorly understood.

Radiocesium contamination effects have differed depending on the habitat condition, and the habitat contamination continues for longer years. To precisely describe the mechanisms of diffusion and the export of cesium-134 and cesium-137 deposited in freshwater streams, the relationship between radiocesium concentration in sand, litter, algae and aquatic invertebrates and the days after diffusion as well as distance from FDNPP were considered in this study. The relationship between the concentration of cesium-134 and cesium-137 in periphytic algae and stream current velocity were also analysed. The effects of water flow on contamination levels in biota were then clarified, and the mechanism of decontamination particularly in areas with low levels of radiocesium contamination area was discussed.

## Materials and methods

### Study site

**Figure 1:**
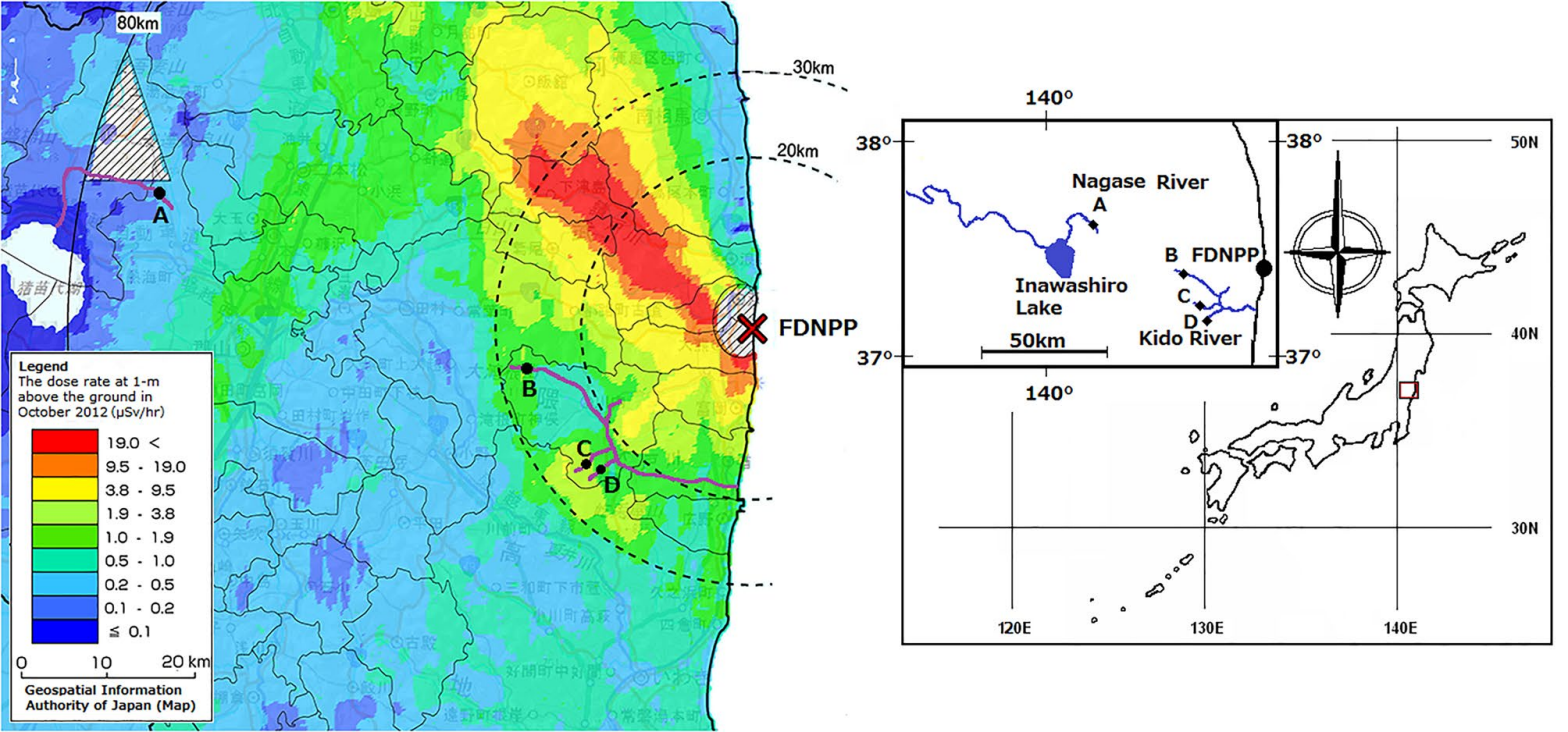
Study site in Fukushima Prefecture, Japan. Square: sampling sites, circle: FDNPP (Fukushima Daiichi Nuclear Power Plant). This map was generated by using software program Microsoft Paint Windows 10.

The study sites were located approximately 20–75 km from the Fukushima Daiichi Nuclear Power Plant in Fukushima Prefecture, Japan (Fig. 1). According to an aircraft radioactivity survey reported by the Ministry of Education, Culture, Sports, Sciences, and Technology of Japan^19^, the air dose rate in this region was 0.3–3.2 μSv/h, and the deposition of cesium-134 and cesium-137 ranged from less than 64,000 to 940,000 Bq/m^2^ (Table 1) in June 2011. The study catchment area is mostly forested and dominated with deciduous trees. Other areas in the region are also forested as well, with Japanese cedar and cypress plantations used for timber production. A field survey was conducted at one headwater tributary (A) of the Nagase River and three headwater tributaries (B, C, and D) of the Kido River. The substrate of these sites was consisted with sand, cobble and rocks. Geological feature of the soil on all the sites was the same, biotite granite. Streams at sites B, C and D were covered with riparian forests and it was difficult for sunlight to penetrate directly. Stream width of site A was wider than sites B, C and D, so sunlight could penetrate through the forest cover and contact the stream surface only along the middle of the stream.

**Table 1:** Air dose rate and the deposition of Cs according to an aircraft radioactivity survey by MEXT (2011), averaged value of dose rate 1-m above the ground on the sampling date from 2013 to 2019 (n = 23) and five environmental factors on four sites on the sampling date from 2013 to 2019 (n = 23).

	A	B	C	D	Kruskal Wallis test (H)
Deposition on June 2011 (KBq/m^2^)	64	174	940	690	–
Dose rate on June 2011 (μSv/h)	0.3	1.1	2.6	3.2	–
Wetted stream width (m)	10	2	3	3	–
Dose rate on sampling date (μSv/h)	0.06 ± 0.021	0.19 ± 0.055	0.41 ± 0.133	0.51 ± 0.145	58.64****
Air temperature (°C)	14.0 ± 5.75	13.7 ± 5.66	16.4 ± 4.39	15.2 ± 6.12	2.56
Water temperature (°C)	10.6 ± 3.69	10.7 ± 3.44	11.6 ± 3.25	10.7 ± 3.81	1.18
pH	7.1 ± 0.39	7.2 ± 0.33	7.2 ± 0.31	7.1 ± 0.37	1.77
Electric conductivity (µs/cm)	52.6 ± 15.48	44.5 ± 15.69	34.4 ± 8.88	31.4 ± 10.13	20.94****
Dissolved oxygen	11.5 ± 0.76	11.2 ± 1.28	11.2 ± 1.07	11.7 ± 1.11	2.11

****P < 0.001.

### Sampling

The air dose rate at 1- m above the ground was measured with a γ survey meter at the sampling site (TCS-172 NaI scintillation counter; ALOKA). The electrical conductivity (EC) of the streams was measured using a portable compact twin conductivity meter (B-173; Horiba); pH was measured using a portable compact twin pH meter (B-212; Horiba), and the dissolved oxygen (DO) was measured using a portable DO meter (DO-5509; Lutron). Stream velocity was measured using a portable meter (V-303, VC-301, KENEK). All parameters were measured at all sites on all sampling dates.

Sand substrate, litter and algae were sampled from stream riffles at a depth of 10-15 cm from July 2013 to April 2019, as was reported in previous studies^13^. The sand substrate was sampled in each riffle to a depth of 5- cm. When sand was not immediately visible in the stream substrate, stones were removed and the sand underneath the stones was sampled. Litter shed in the water was collected after gentle hand-rinsing. Leaf litter forms the base of stream food webs. Periphytic algae were collected by brushing the pebbles or rocks with a toothbrush. These algae are also primary producers at the base of stream food webs. Prior to brushing, we gently hand-rinsed the stone surface to remove other organic matter and aquatic invertebrates in the periphyton.

Aquatic invertebrates from thirteen groups (*Perlidae Gen.* spp., Nemouridae *Gen.* spp., *Ephemera japonica*, *Ephemerellidae Gen.* spp., *Heptageniidae Gen.* spp., *Hydropsychidae Gen.* spp., *Stenopsychi spp*., *Rhyacophilidae Gen.* spp., *Epiophlebia superstes*, *Lanthus fujiacus*, *Tipulidae Gen.* spp., and *Corydalidae Gen.* spp., *Geothelphusa dehaani*,) were qualitatively sampled from riffles at a depth of 10-15 cm at the four sites from July 2013 to April 2019. At each site, a D-frame net with a 1-mm mesh was placed downstream of the sampling area on the substrate in water. We then disturbed the substrate upstream of the net, allowing insects to drift into the D-frame net. The sampled aquatic invertebrates were identified to family level in the field and then frozen.

Three bricks (210 × 100 × 60 mm) were placed separately within the stream riffle at a depth of 10–20 cm on August 25, 2014 at each of the four sites. Then, periphytic algae growing on the bricks were collected by brushing the substrate with a toothbrush. Before brushing, we gently hand-rinsed the brick surface in running water to remove other organic matter from the periphytic algae. The sampling was carried out eight times: in October and December 2014; March, May, June, July and November 2015; and April 2016. Stream velocity of right side, upper reaches side and left side of each brick were measured and averaged. This averaged value was used as the stream velocity of each periphytic algae sample.

### Radiocesium analysis

Radiocesium was analysed according to the methods in previous studies^10,20^. Samples of sand substrate and litter were dried at 75 °C in an oven. Thereafter, samples of sand were placed in a sieve (mesh size 2 mm; Iida, Japan), and the sand that passed through the sieve was used, meaning that the sand substrate in this study included silt granules. Samples of algae were concentrated via evaporation and dried in an oven at 75 °C. Samples of aquatic invertebrates were also dried in an oven at 75 °C. All samples were homogenized and packed into 100-ml polystyrene containers (U-8). Gamma-ray spectrometric measurements were performed on each sample. The radioactive concentrations of cesium-134 (604 keV) and cesium-137 (662 keV) were measured using an HPGe coaxial detector system (GEM40P4-76, Seiko EG and G, Tokyo, Japan) at the Forestry and Forest Products Research Institute (FFPRI) with a time of 36,000 s or longer. Data with a standard error of < 10% were used for analysis. Gamma-ray peaks at 604.66 and 661.64 keV were used to identify cesium-134 and cesium-137, respectively. The measurement system was calibrated using a standard gamma-ray source (MX033U8PP; Japan Radioisotope Association, Tokyo, Japan), and a standard soil material (IAEA-444) was used to check the measurement accuracy (relative expanded uncertainty value: 4.6%).

The homogenized samples of aquatic invertebrates were further homogenized with Na_2_SO_4_ for radiocesium analysis. The processed aquatic invertebrates were packed into 20-ml plastic tubes. Gamma-ray spectrometric measurements were performed on each tube. Total concentrations of cesium-134 and cesium-137 were measured using a gamma particle counter with a NaI crystal detector (2480WIZARD2, Perkin Elmer, Downers Grove, IL, USA) and a sampling time of 21,600 s at the FFPRI. The measurement system was calibrated with a standard gamma-ray source (E-265/107F16-3, Canberra Industries, Oak Ridge, TN, USA) and a standard soil material (IAEA-444) was used to check the measurement accuracy (relative expanded uncertainty value: 2.9%). A strong correlation between the data obtained with the HPGe coaxial detector system and the data obtained with the NaI crystal detector was observed (cesium-134: τ = 0.75, z = 5.61, n = 33, *P* < 0.001; cesium-137: τ = 0.76, z = 8.43, n = 63, *P* < 0.001; Kendall test). As the detection limit of the HPGe coaxial detector system was higher than that of the gamma particle counter with the NaI crystal detector, the data obtained with the gamma particle counter with the NaI crystal detector were used for the analysis of aquatic invertebrates because we could then use smaller numbers of aquatic invertebrates. Only samples with a dry mass > 50 mg were included in the analysis to avoid measurement errors due to the low quantity.

### The relation between radiocesium concentration and the days after accident

The data measured for sand substrate, litter, algae and aquatic invertebrates were plotted on a logarithmic scale against the days after accident occurred, and correlation coefficients were calculated. The significance of the correlations was statistically assessed using the Pearson’s product-moment correlation coefficients. Radiocesium concentrations that were below the detection level on a dry weight basis because of insufficient mass for radiocesium analysis were excluded from the analysis. Using the regression curves, the radiocesium decay was corrected to September 1, 2013 and the differences in the values among sites were statistically tested using the Friedman-test.

### The ecological half-life and the transfer coefficient

Ecological half-life was estimated by fitting the measured data to an exponential curve with the equation:

$${\text{Ln}}\,{\text{Y}} = {\text{Ln}}\,{\text{A}}{-}{\text{Ln}}\,2*\left( {{\text{t}}/{\text{T}}_{{{\text{ecol}}}} } \right),$$

where the activity concentration in a sample group is plotted as a function of time. A is a constant, T_ecol_ is the ecological half-life, and t is time after the accident. The ecological half-life of cesium-134 and cesium-137 was estimated through exponential curve fitting. A positive relationship between the radiocesium concentration and the days after the accident indicates the increase of radiocesium according to the time, thus the ecological half-life cannot be calculated under this scenario. It would be better to include the physical decay in calculations of the biological half-life using long-term data^21^. However the research of this study started 2 years after the accident and the research period was only 6 years. Moreover, we did not need to establish the biological half-life, but rather the ecological half-life. For this reason, in this study, we did not consider the physical decay of a radionuclide to calculate the ecological half-life, which enabled us to integrate the actual radionuclide transition in the field. Differences in ecological half-life among sites were statistically tested using the Friedman-test.

The transfer coefficient (T) is an empirical coefficient that can be used in predictive models for natural ecosystems. It is defined by the following expression:

$${\text{T}} = {\text{Activity}}\,{\text{concentration}}\,{\text{of}}\,{\text{the}}\,{\text{invertebrate}}\,{\text{samples}}\,\left( {{\text{Bq}}/{\text{kg}}} \right)/{\text{Activity}}\,{\text{concentration}}\,{\text{of}}\,{\text{the}}\,{\text{algae}}\,{\text{or}}\,{\text{litter}}\,\left( {{\text{Bq}}/{\text{kg}}} \right)$$

The diet of some aquatic invertebrate species comprises litter rather than algae, whereas the diet of other species comprises both algae and litter. In this analysis, all the invertebrate groups were analysed under the assumption that their diets were dependent on algae or litter. Differences in the T value among sites were statistically tested using the Friedman-test.

### The relation between radiocesium concentration and current velocity

The radiocesium concentrations based on dry weight were estimated for September 1, 2013. Radiocesium concentrations that were below the detection level on a dry weight basis, because of insufficient mass for radiocesium analysis, were excluded from statistical analysis. Nonparametric tests for statistical analysis were conducted. The Kendall rank correlation test was used to clarify the relationships between stream velocity and concentrations of radioactive cesium-134 and cesium-137. To examine the differences in the decline between sites A and D, the comparison of two regression slopes test was conducted.

## Results

The dose rates in the air in June 2011 at sites C and D were higher than those at sites A and B. The deposition of radiocesium in June 2011 followed the same pattern. There were no differences in values for air or water temperature, pH, or DO among the four sites, whereas the EC and dose rate on the sampling dates differed significantly among sites (Table 1).

The estimated cesium-134 and cesium-137 concentrations on September 1, 2013 differed depend on the groups and sampling sites. The radiocesium concentration was higher in *Ephemera japonica* and *Lanthus fujiacus* and lower in *Perlidae Gen.* spp. and *Corydalidae. Gen.* spp. (Table 2). The radiocesium concentration was significantly lower at site A and higher at sites C and D (Cesium-134: χ^2^ = 19.3, P < 0.001; Cesium-137: χ^2^ = 20.5, P < 0.001; Friedman-test, Table 2).

**Table 2:** Estimated radiocesium concentration using regression curve (R) and the correlation coefficient between the cesium-134 and ccesium-137 concentrations and the years after accident in 16 groups (C) and the number of samples tested (N) at A, B, C and D sites.

	Site A			Site B			Site C			Site D		
	R	C	N	R	C	N	R	C	N	R	C	N
**Cesium-134**												
Sand (include silt)	35	− 0.35	20	226	− 0.89***	25	289	− 0.86***	18	346	− 0.96***	20
Litter	104	− 0.60***	26	415	− 0.55***	30	816	− 0.67***	34	986	− 0.67***	29
Algae	245	− 0.40*	23	574	− 0.54***	30	896	− 0.20	32	692	− 0.08	25
*Perlidae Gen.* spp.	21	**0.13**	9	83	− 0.29	8	151	− 0.30	13	81	− 0.34	9
*Nemouridae Gen.* spp.	None	None	0	109	− 1.00***	2	636	− 0.45	5	512	− 0.62	4
*Ephemera japonica*	228	− 0.99**	3	237	− 0.33	20	953	− 0.73**	12	1675	− 0.93***	9
*Ephemerellidae Gen.* spp.	28	− 0.07	13	193	− 0.94**	6	190	− 0.36	7	426	− 0.53	4
*Heptageniidae Gen.* spp.	36	**0.04**	9	65	− 0.55	9	377	− 0.57*	13	281	− 0.65	6
*Hydropsychidae Gen.* spp.	35	**0.42**	5	231	− 0.58	8	238	− 0.40	16	283	− 0.50	8
*Stenopsyche* spp.	95	− 0.79*	7	315	− 0.73	5	836	− 0.98***	6	None	None	0
*Rhyacophilidae Gen.* spp.	183	− 0.81	4	94	− 0.44	5	82	0.97**	4	2285	− 0.47	4
*Epiophlebia superstes*	58	1.00***	2	124	− 0.47	7	270	− 0.98***	5	144	− 0.68	5
*Lanthus fujiacus*	419	− 1.00***	2	212	− 0.28	7	2655	− 1.00***	2	404	− 0.98**	4
*Tipulidae Gen.* spp.	117	− 0.61	6	83	0.67*	8	290	− 0.19	10	144	− 0.78*	6
*Corydalidae. Gen.* spp.	54	− 0.76	4	50	**0.80**	4	148	− 0.43	3	None	None	0
*Geothelphusa dehaani*	None	None	0	505	− 0.58	3	228	**0.02**	10	831	− 0.54	6
**Cesium-137**												
Sand (include silt)	83	**0.32**	20	495	− 0.68**	25	578	− 0.45*	19	783	− 0.75***	20
Litter	238	− 0.46**	31	975	− 0.36*	37	1854	− 0.50***	40	2228	− 0.46**	33
Algae	518	− 0.26	23	1126	− 0.58***	33	2096	− 0.14	37	2774	− 0.35	33
*Perlidae Gen.* spp.	46	**0.47**	9	180	− 0.14	8	412	− 0.32	15	227	− 0.47	9
*Nemouridae Gen.* spp.	None	None	0	237	1.00***	2	3457	− 0.81*	5	1112	− 0.54	4
*Ephemera japonica*	495	− 0.97*	3	489	− 0.04	21	1224	− 0.21	14	2750	− 0.64*	10
*Ephemerellidae Gen.* spp.	62	**0.12**	13	410	− 0.57	7	564	− 0.56	8	797	− 0.77	5
*Heptageniidae Gen.* spp.	78	**0.36**	9	140	− 0.09	9	986	− 0.63*	14	560	− 0.44	7
*Hydropsychidae Gen.* spp.	77	**0.50**	5	641	− 0.71*	9	664	− 0.31	17	811	− 0.50	8
*Stenopsyche* spp.	207	− 0.56	7	734	− 0.49	8	1113	− 0.53	7	None	None	0
*Rhyacophilidae Gen.* spp.	396	− 0.73	4	203	− 0.30	5	177	0.98**	4	3314	− 0.93**	5
*Epiophlebia superstes*	125	1.00***	2	372	− 0.58	6	818	− 0.98***	5	312	− 0.51	5
*Lanthus fujiacus*	908	− 1.00***	2	461	**0.04**	7	5775	− 1.00***	2	786	− 0.97**	4
*Tipulidae Gen.* spp.	253	− 0.31	6	181	0.76*	8	848	− 0.34	11	397	− 0.89**	6
*Corydalidae. Gen.* spp.	117	− 0.74	4	109	0.89**	4	321	− 0.04	3	None	None	0
*Geothelphusa dehaani*	None	None	0	1095	− 0.48	3	494	**0.24**	10	2127	− 0.59	7

R: Estimated radiocesium concentration on 1 September, 2013 by the equation (Bq/kg of dry weight), C: correlation coefficient, N: number of sampling data, Bold letter: positive relation, *:0.05, **:0.01, ***:0.001.

Radiocesium concentrations in sand substrates at sites B, C and D significantly decreased with the years, but not at A. The radiocesium concentrations in litter significantly decreased with the years at the four sites. The radioccesium concentrations in algae decreased with the years and the relation was significant at site B. Radiocesium concentration in aquatic invertebrates at site D decreased with the years, and significant decreases were observed in *Ephemera japonica*, *Lanthus fujiacus*, and *Tipulidae Gen*. spp. for cesium-134 and *Ephemera japonica*, *Rhyacophilidae Gen.* spp., *Lanthus fujiacus*, and *Tipulidae Gen*. spp. for cesium-137. The radiocesium concentrations at site C also decreased with the years and the decreases were significant in *Ephemera japonica*, *Heptageniidae Gen.* spp., *Stenopsyche* spp., *Epiophlebia superstes*, and *Lanthus fujiacus* for cesium-134 and *Nemouridae Gen*. spp., *Heptageniidae Gen.* spp., *Epiophlebia superstes*, and *Lanthus fujiacus* for cesium-137, except for those in *Rhyacophilidae Gen.* spp. and *Geothelphusa dehaani* that increased. The radiocesium concentrations in aquatic invertebrates at site B decreased with the years and the decreases were significant in *Nemouridae Gen.* spp. and *Ephemerellidae Gen.* spp. for cesium-134 and *Hydropsychidae Gen.* spp. for cesium-137, except for those in *Nemouridae Gen.* spp., *Lanthus fujiacus*, *Tipulidae Gen.* spp., *Corydalidae Gen.* spp., which showed a positive relationship. No significant decreases in radioccesium concentrations were observed at site A except for those in *Ephemera japonica* and *Lanthus fujiacus* (Table 2).

The ecological half-life of cesium-134 was 0.3–13.1 years, and that of cesium-137 was 0.3–52.6 years; the half-life varied by groups and sites (Table 3). Because the relationship between the radiocesium concentration and the year was positive in some groups (Table 2), ecological half-life could not be calculated for those groups. The positive relationship between the radioesium concentration and the year indicates a longer ecological half-life, and positive relationship was often observed at site A. The ecological half-life was longer where the air dose rate was lower (cesium-134: χ^2^ = 19.3, P < 0.001; cesium-137: χ^2^ = 20.5, P < 0.001; Friedman-test).

**Table 3:** Ecological half-life of 16 groups at sites A, B, C and D.

	A Years ± 95% CI	B Years ± 95% CI		C Years ± 95% CI			D Years ± 95% CI	
**Cesium-134**								
Sand (include silt)	8.1 ± 0.03	2.4 ± 0.13		2.7 ± 0.18			3.0 ± 0.11	
Litter	2.1 ± 0.17	3.3 ± 0.37		2.8 ± 0.68			2.5 ± 1.13	
Algae	1.8 ± 0.28	1.9 ± 0.13		3.7 ± 0.82			8.4 ± 0.73	
*Perlidae Gen.* spp.	–	3.3 ± 0.26		8.5 ± 0.19			5.1 ± 0.16	
*Nemouridae Gen.* spp.	None	8.0		9.0 ± 0.57			0.9 ± 0.53	
*Ephemera japonica*	2.3 ± 0.02	3.4 ± 0.76		1.4 ± 0.66			1.8 ± 1.05	
*Ephemerellidae Gen*. spp.	13.1 ± 0.10	2.4 ± 0.08		3.1 ± 0.19			1.7 ± 0.49	
*Heptageniidae Gen*. spp.	–	3.9 ± 0.08		2.2 ± 0.40			2.3 ± 0.99	
*Hydropsychidae Gen*. spp.	–	3.8 ± 0.25		4.5 ± 0.29			4.9 ± 0.57	
*Stenopsyche* spp.	2.2 ± 0.10	3.2 ± 0.26		1.7 ± 0.16			None	
*Rhyacophilidae Gen*. spp.	1.1 ± 0.23	1.7 ± 0.20		–			0.8 ± 0.80	
*Epiophlebia superstes*	–	8.5 ± 0.14		1.8 ± 0.11			1.7 ± 0.21	
*Lanthus fujiacus*	2.1	5.3 ± 0.17		1.6			1.8 ± 0.07	
*Tipulidae Gen.* spp.	2.7 ± 0.31	–		6.3 ± 1.00			3.3 ± 0.11	
*Corydalidae. Gen.* spp.	0.3 ± 0.24	–		4.2 ± 0.81			None	
*Geothelphusa dehaani*	None	1.1 ± 0.89		–			3.3 ± 1.49	
**Cesium-137**								
Sand (include silt)	–	5.9 ± 0.30		9.2 ± 0.41			8.3 ± 0.22	
Litter	3.1 ± 0.40	8.4 ± 0.85		6.8 ± 1.25			5.2 ± 2.28	
Algae	3.0 ± 0.70	3.9 ± 0.43		11.7 ± 2.63			4.7 ± 2.34	
*Perlidae Gen.* spp.	–	11.8 ± 0.57		7.6 ± 0.67			4.7 ± 0.33	
*Nemouridae Gen.* spp.	None	–		2.8 ± 2.17			1.1 ± 1.79	
*Ephemera japonica*	4.4 ± 0.13	34.3 ± 1.65		11.3 ± 1.68			5.4 ± 2.83	
*Ephemerellidae Gen*. spp.	–	4.5 ± 0.29		3.8 ± 0.71			3.6 ± 1.20	
*Heptageniidae Gen*. spp.	–	27.0 ± 0.21		2.8 ± 1.07			6.2 ± 1.83	
*Hydropsychidae Gen*. spp.	–	3.1 ± 0.65		6.3 ± 1.03			4.1 ± 1.61	
*Stenopsyche* spp.	4.2 ± 0.25	6.1 ± 0.90		6.0 ± 0.76			None	
*Rhyacophilidae Gen*. spp.	1.4 ± 0.50	2.6 ± 0.45		–			1.5 ± 2.03	
*Epiophlebia superstes*	–	2.9 ± 0.52		1.5 ± 0.26			2.6 ± 0.48	
*Lanthus fujiacus*	3.9	–		2.4			4.2 ± 0.25	
*Tipulidae Gen.* spp.	6.3 ± 0.72	–		5.0 ± 2.50			2.8 ± 0.24	
*Corydalidae. Gen.* spp.	0.3 ± 0.52	–		52.6 ± 2.22			None	
*Geothelphusa dehaani*	None	1.4 ± 2.73		–			4.2 ± 3.87	

CI: confidence interval; –: positive relation between the cesium-134 and cesium-137 and the year; none: no sample.

The transfer coefficient values were mostly below 1 and differed significantly among sites based on algae (cesium-134: χ^2^ = 14.7, P < 0.005; cesium-137: χ^2^ = 17.4, P < 0.001; Friedman test) and on litter (cesium-134: χ^2^ = 18.6, P < 0.001; cesium-137: χ^2^ = 19.9, P < 0.001; Friedman test), and higher value was shown at site A. Transfer coefficient for *Ephemera japonica* and *Lanthus fujiacus* tended to be higher. Transfer coefficient for *Perlidae Gen.* spp. and *Corydalidae Gen.* spp. tended to be lower (Tables 4, 5).

**Table 4:** Transfer coefficient in 13 groups of aquatic invertebrates at sites A, B, C and D.

Sites	Cesium-134				Cesium-137			
	A	B	C	D	A	B	C	D
*Perlidae Gen.* spp.	0.24	0.17	0.16	0.16	0.25	0.16	0.18	0.16
*Nemouridae Gen.* spp.	–	0.55	0.60	0.40	–	0.49	0.44	0.37
*Ephemera japonica*	1.11	0.52	0.48	1.17	1.14	0.50	0.45	1.10
*Ephemerellidae Gen.* spp.	0.46	0.46	0.14	0.48	0.45	0.43	0.16	0.29
*Heptageniidae Gen.* spp.	0.57	0.19	0.19	0.22	0.54	0.19	0.20	0.29
*Hydropsychidae Gen.* spp.	0.78	0.61	0.24	0.26	0.75	0.52	0.25	0.31
*Stenopsyche* spp.	0.86	0.76	0.24	–	0.74	0.71	0.24	–
*Rhyacophilidae Gen.* spp.	0.55	0.27	0.31	0.40	0.46	0.25	0.31	0.40
*Epiophlebia superstes*	0.83	0.35	0.11	0.13	0.80	0.30	0.10	0.12
*Lanthus fujiacus*	2.18	0.55	0.41	0.17	2.25	0.51	0.44	0.38
*Tipulidae Gen.* spp.	0.52	0.34	0.20	0.18	0.53	0.31	0.18	0.15
*Corydalidae. Gen.* spp.	0.13	0.23	0.17	–	0.12	0.21	0.16	–
*Geothelphusa dehaani*	–	0.74	0.40	0.84	–	0.73	0.40	0.88

**Table 5:** Transfer coefficient from litter in 13 groups of aquatic invertebrates at sites A, B, C and D.

Sites	Cesium-134				Cesium-137			
	A	B	C	D	A	B	C	D
*Perlidae Gen.* spp.	0.54	0.30	0.38	0.32	0.56	0.31	0.36	0.20
*Nemouridae Gen.* spp.	–	0.33	1.19	0.60	–	0.32	0.82	0.64
*Ephemera japonica*	2.83	0.96	0.93	0.93	2.81	0.97	0.94	0.85
*Ephemerellidae Gen.* spp.	0.89	0.49	0.31	0.25	0.92	0.51	0.28	0.23
*Heptageniidae Gen.* spp.	0.86	0.17	0.31	0.20	0.98	0.16	0.30	0.20
*Hydropsychidae Gen.* spp.	1.09	0.53	0.60	0.43	1.06	0.47	0.57	0.36
*Stenopsyche* spp.	1.21	0.53	0.60	–	1.25	0.52	0.61	–
*Rhyacophilidae Gen.* spp.	1.39	0.21	0.64	0.82	1.41	0.19	0.61	0.66
*Epiophlebia superstes*	1.83	0.64	0.20	0.14	1.59	0.35	0.19	0.14
*Lanthus fujiacus*	4.99	0.65	0.72	0.39	5.77	0.65	0.78	0.42
*Tipulidae Gen.* spp.	3.24	1.24	0.59	0.18	3.00	1.29	0.53	0.19
*Corydalidae. Gen.* spp.	0.62	0.39	0.24	–	0.59	0.40	0.25	–
*Geothelphusa dehaani*	–	0.79	0.49	0.77	–	0.79	0.49	0.78

–: no sample.

**Figure 2:**
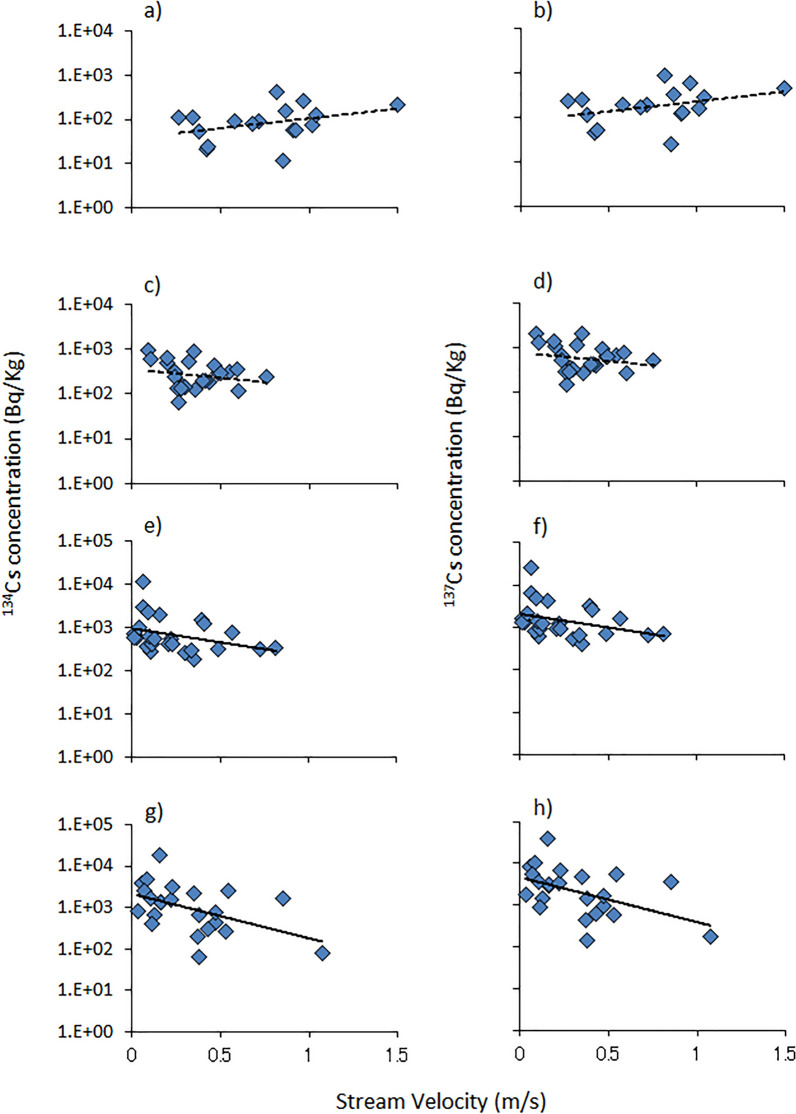
Relation between stream velocity and the radiocesium concentrations (cesium-134, cesium-137) of algae. Solid line: significant correlation, dotted line: trend towards significance. a) site A, cesium-134; b) site A, cesium-137; c) site B, cesium-134; d) site B, cesium-137; e) site C, cesium-134; f) site C, cesium-137; g) site D, cesium-134; h) site D, cesium-137.

The cesium-134 and cesium-137 concentrations in the algae at sites C and D decreased significantly with the increase in stream velocity (cesium-134: site C: τ =  − 0.30, z =  − 2.13, n = 25, *P* < 0.05, Fig. 2e; site D: τ =  − 0.31, z =  − 2.07, n = 23, *P* < 0.05, Fig. 2g; cesium-137: site C: τ =  − 0.30, z =  − 2.13, n = 25, *P* < 0.05, Fig. 2f; site D: τ =  − 0.30, z =  − 2.02, n = 23, *P* < 0.05, Fig. 2h; Kendall test). The cesium-134 and cesium-137 concentrations of algae at site B tended to decrease with the increase in stream velocity, but it was not significant (cesium-134: τ =  − 0.11, z =  − 0.77, n = 25, n.s., Fig. 2c; cesium-137: τ =  − 0.11, z =  − 0.77, n = 25, n.s., Fig. 2d; Kendall test). The cesium-134 and cesium-137 concentrations in algae at site A tended to increase with the stream velocity, but this was also not significant (cesium-134: τ = 0.21, z = 1.16, n = 19, n.s., Fig. 2a; cesium-137: τ = 0.21, z = 1.15, n = 19, n.s., Fig. 2b; Kendall test).

We calculated the slope coefficient between stream velocity and concentrations of cesium-134 and cesium-137 as a logarithmic function and examined the differences in coefficients between the site A, which has the lowest average air dose rate and site D, which has the highest average air dose rate by comparing the regression slopes of the regressions of the resulting plots. The slopes differed significantly between sites (cesium-134: t = 2.84, n = 39, *P* < *0.01*; cesium-137: t = 2.83, n = 39, *P* < *0.01*; comparison of two regression slopes test).

## Discussion

The algae attached to brick surfaces in the low-velocity riffle had higher radiocesium concentrations than those in the high-velocity riffle at sites C and D. Therefore, in a contaminated area, more cesium-134 and cesium-137 would be taken up into the algal mat in a low-velocity area compared with in a high-velocity area. The radiocesium in stream water exists in both dissolved and suspended forms^22^ Dissolved and suspended radiocesium can easily penetrate the algal mat, thus, the intake of radiocesium within the algal mat would occur regardless of the form of radiocesium. Consequently, the involvement of radiocesium in the algal mat would easily occur at low water velocities. Of course, cesium-134 and cesium-137 are also trapped in the litter mat and sand substrate. Considering this result, aquatic insects that consume the algae mat as part of their diet^15^ and fish that consume the aquatic insects as part of their diet, which inhabit low-velocity areas, would also exhibit higher radiocesium concentrations via the food web.

Yoshimura and Akama^10^ found no significant differences in the radiocesium levels in algae and aquatic insects between sites where the air dose rates differed (0.08–0.13 and 0.23–0.28 μSv/h) and also reported high variability in radiocesium levels in algae and aquatic insects (range: ca.300 Bq/kg). This may be due, in part, to the difference in the assemblages of the algae species attached to the stones at the sites. However, considering our results, the variability in the radiocesium concentration of algae might be due primarily to differences in the velocities of the streams where the algae were attached (ca: 500–5000 Bq/kg depend on the velocity at site D). The variability caused by the differences in stream velocity was sufficiently large to obscure the differences in contamination levels at sites with different deposition. Subsequently, the radiocesium concentration in aquatic insects that graze on algae would also show variability. Stream velocity plays a large role in habitat contamination, and might contribute to the variation in radiocesium concentration in the freshwater biota.

Particulate organic matter from the surrounding forest, which is contaminated with radiocesium, settles to the stream bottom when the flow velocity is low^23–27^ and releases radiocesium at the stream bed when it decomposes^28–30^. The lack of flow in deep parts of a pool can also result in the accumulation of silt contaminated with radiocesium and free radiocesium in stagnant water. Most of the radionuclides in a pond were bound to the sediment, and aquatic insects in the pond acquire radionuclides from the sediment and ingested biotic components^14^. Consequently, fish living near the bottoms of lakes ingest material that is highly contaminated with radiocesium that had settled on the bottom. The higher concentrations of cesium-137 in brown trout (*Salmo trutta*) in lakes versus streams^20^ suggest that the differences in contamination level are due to differences in the water flow rate. In freshwater charr (*Salvelinus leucomaenis*), there is substantial variation in the cesium-137 levels in equal-aged fish in a given stream^20^. Habitat selection by individual fish might cause variation in radiocesium concentrations in the diet, and levels of contamination in the diet can differ with stream velocity (habitat), even if the diet is the same. These factors would enhance the variability in radiocesium concentration in equal-aged fish inhabiting the same area.

The transfer coefficient differed among aquatic invertebrate groups. This value was calculated on the assumption that all of the aquatic invertebrates ate algae or litter. However, the diet of all these aquatic invertebrates is not restricted only to algae or litter. Because the concentration in the litter was a little lower than that in the algae, the transfer coefficient based on litter would also be a little higher than that based on algae. Some groups such as *Perlidae Gen.* spp. are predators. However, the transfer coefficient for the predators did not largely differ from the other aquatic invertebrates, because the average radiocesium values in aquatic invertebrates were similar to those in algae and litter. Based on the differences in radiocesium values of the diet, the transfer coefficient calculated in this study also did not differ greatly from the actual value.

The ecological half-life of cesium-137 ranges from 0 to more than 10 years depending on the sample type^31,32^. The value of ecological-half life in this study were largely within this range although the sample types differed. The transfer coefficient differed among aquatic invertebrate groups. Because the algae in this study were sampled from the stones in the riffle, the aquatic invertebrate groups with higher transfer coefficient would inhabit the place where radiocesium tends to be accumulated such as the low stream-velocity areas. Because the habitats of these groups with higher transfer coefficients were contaminated with radiocesium, they could not effectively exclude the radiocesium from the body. In fact, *Ephemera japonica* and *Lanthus fujiacus* inhabit low stream-velocity areas^15^. Estimated radiocesium concentrations also showed higher va in these groups. On the other hand, some other groups with lower transfer coefficients, such as *Perlidae Gen.* spp. and *Corydalidae Gen.* spp. inhabit the high stream-velocity area^15^ where radiocesium does not tend to be accumulated.

The significantly higher cesium-134 and cesium-137 concentrations in algae in streams with low velocities was detected only at sites C and D, which were heavily contaminated areas. At sites A and B, which were less contaminated with radiocesium, no decline correlation between the radiocesium concentration and stream velocity was observed. Besides, at site A, the concentration tended to be higher at rapid stream velocities. Significant difference in the degree of slope coefficient between sites A and D would indicate that radiocesium dynamic are different between more and less contaminated areas.

**Figure 3:**
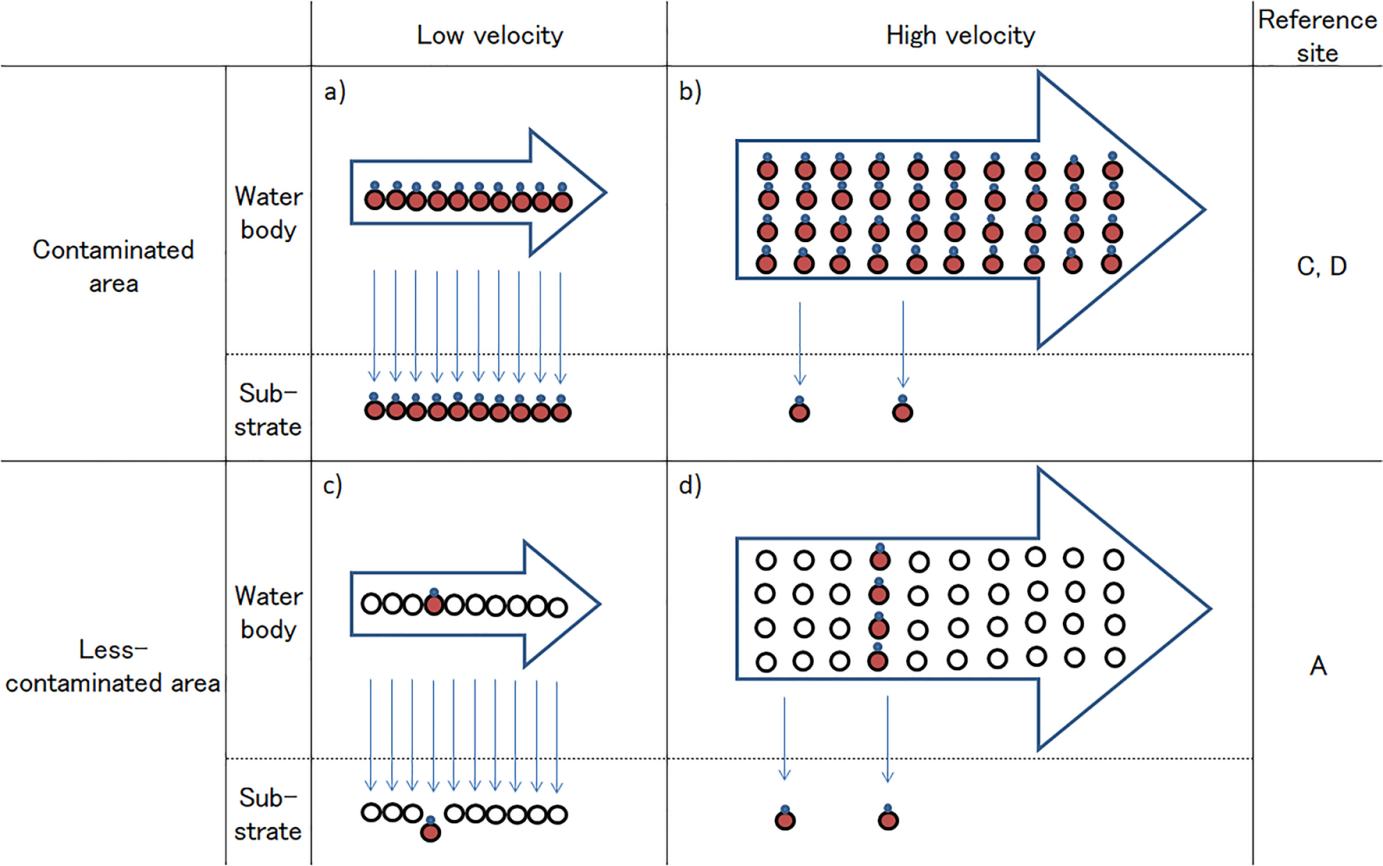
Mimetic diagram of the settling pattern to the bottom of the stream. Large circle: silt grain, small circle: radioactive Cs, a) low velocity in contaminated area, b) high velocity in contaminated area, c) low velocity in less-contaminated area, d) high velocity in less-contaminated area.

In general, more sand and silt material is transported to high-velocity area than the low-velocity area of the stream because there is a higher volume of water in the former areas. However, in high-velocity area, most of the sand and silt passes through the stream without settling to the bottom of the stream. Because the air dose rate was higher at sites C and D, most of the silt grains might have been contaminated with radiocesium at sites C and D. Indeed, radiocesium amounts in the sand substrate (including silt) at sites C and D were higher than that at site A. Figure 3 shows the pattern diagram of sand settling to the stream bottom in highly radiocesium contaminated areas and less radiocesium contaminated areas. A rapid stream velocity would reduce the effect of contamination in algae (Fig. 3b) compared with the slow velocity (Fig. 3a). However, in the less contaminated areas, the phenomenon would result in a different outcome.

Generally, an increase in particle diameter leads to an increase in settling velocity in water^33,34^. An increase in weight also leads to an increase in settling velocity in water under the influence of gravity. Materials that are attached with substances such as radiocesium should be heavier and larger than unattached particles. Therefore, materials contaminated with radiocesium should sink to the bottom of the stream faster than uncontaminated materials^35,36^. In general, the percentage of silt and sand materials contaminated with radiocesium in the flow is not different between high- and low-velocity areas because they are connecting and form one stream. More silt materials are transported to the flow in the high-velocity areas than the low-velocity areas because of the higher volume of water in the high-velocity area. Thus, the number of silt grains with radiocesium that sink to the bottom would be greater in the high stream-velocity areas (Fig. 3c, d), because contaminated materials are heavier and larger than the uncontaminated materials. Consequently, the radiocesium concentrations in the algae mat in the high-velocity areas tended to be higher than those in the low-velocity areas in the less-contaminated area.

Some of the aquatic invertebrate groups exhibited a positive relationship between radiocesium concentration and days, particularly at site A where the air dose rate was lower. As radiocesium concentration was low at site A, the variation in radiocesium concentration, which is dependent on environmental factors, was large enough to obscure any decrease in the concentration, and thus decreases were not detected for a few years. However, considering from the mechanism of radiocesium-contaminated silt grains accumulation at site A, a positive relationship between radiocesium concentration and the days after the accident would indicate that the ecological half-life should be far longer. Higher transfer coefficients at site A also indicate that radiocesium concentration in aquatic invertebrates were higher compared to those in algae. A continuous decrease in the radiocesium concentration would be obvious in more contaminated areas, but it is difficult to reduce the radiocesium concentration further when it has already reached a low level due to the alternation among contamination mechanisms.

Radiocesium is transported by the continuing input of radioactive substances from the catchment, and the radiocesium then would settle to the bottom of the stream. The radiocesium in stream water exists in dissolved and suspended forms^22^. The dissolved cesium-134 and cesium-137 in running water is largely not adsorbed by soil and is also readily taken up by microbes, algae and plants. This transport pathway of cesium-134 and cesium-137 eventually facilitates the uptake by aquatic invertebrates and freshwater fish at higher trophic levels in the food web. In this study, transport and transfer of Cs associated particles was discussed without considering the effects of irreversible and reversible sorption of Cs. These factors would also be essential for uptake in the biota. However, physiological experiments would be needed individually in order to fully understand these factors, and these will be the subject of future research. Difference in accumulation mechanisms between low and high velocity areas would lead to longer periods of contamination. Radiocesium is considered to be accumulated in some places such as pool or low stream-velocity area and it would also continuously affect the riffle stream substrate, especially in areas with lower concentration of radiocesiums. The transfer of radiocesium in aquatic ecosystems is also affected by other factors such as pH, temperature and dissolved organic carbon because the physiological function differs depend on the pH and water temperature^37–39^. Speciation of cesium and the concentration of other ions also affect the cesium transfer in the body^40–42^. Fishes are major functional components of forest stream ecosystems^43,44^. Radioactive contamination in fish should be avoided because they may be a source of food for human. A safety threshold of 100 Bq/kg of radiocesium was introduced in April 2012, but activity concentrations greater than this have been detected in fish hundreds of kilometres away from the FDNPP^20^. Clearly, it is critical to reduce radiocesium food contamination. The volume of water flow due to disturbances and stream velocity are key determinants of future contamination in freshwater ecosystems. Monitoring and continuous decontamination, even if the radiocesium concentration is low, are also necessary for the places where contamination levels have reached to a lower threshold.
